# Perspectives from Supplemental Nutrition Assistance Program Participants on Improving SNAP Policy

**DOI:** 10.1089/heq.2018.0094

**Published:** 2019-03-21

**Authors:** Cindy W. Leung, Julia A. Wolfson

**Affiliations:** ^1^ Department of Nutritional Sciences, University of Michigan School of Public Health, Ann Arbor, Michigan.; ^2^ Department of Health Management and Policy, University of Michigan School of Public Health, Ann Arbor, Michigan.

**Keywords:** food insecurity, health promotion, nutrition policy, population health, poverty

## Abstract

**Purpose:** Supplemental Nutrition Assistance Program (SNAP) participants are important stakeholders in improving program policies, but their voices have not been included in the public discourse.

**Methods:** We assessed the opinions of 202 SNAP participants and 368 food-insufficient nonparticipants on proposed SNAP policies.

**Results:** The majority of SNAP participants and nonparticipants supported increasing federal SNAP spending, policies to broaden the usage of SNAP benefits, and policies to improve the healthfulness of foods purchased with SNAP benefits. However, 60% opposed the America's Harvest Box proposal.

**Conclusion:** The perspectives of SNAP participants on restructuring the program's reach or nutritional impact should be considered.

## Introduction

The Supplemental Nutrition Assistance Program (SNAP) is the nation's largest safety net protecting low-income individuals from poverty and hunger.^1^ In 2018, SNAP served 40.6 million individuals at a total cost of $51 billion.^2^ SNAP has been subjected to much debate about policies to change the program's administration or its nutritional impact. As an example, in early 2018 as part of the 2018 Farm Bill debate, the U.S. Department of Agriculture (USDA) unveiled “America's Harvest Box,” a proposal that would replace half of SNAP benefits with a box of shelf-stable foods, impacting the majority of SNAP households.^3^ To inform the national SNAP discussion, this study assessed the opinions of SNAP participants and food-insufficient nonparticipants on federal SNAP spending, policies to change the program's eligibility or nutritional impact, and the Harvest Box proposal. SNAP participants were included in the study because of their in-depth experience with the program's current policies and because they would be the group most impacted by any policy changes. Nonparticipants who report experience of food insufficiency were included in the study because they represent individuals who might have participated in SNAP in the past and individuals who may apply for SNAP in the future.

## Materials and Methods

### Study population

Respondents were recruited using TurkPrime, an independent platform for researchers that integrates with MTurk to facilitate data collection for social sciences research.^4^ In brief, researchers can recruit respondents by posting a survey as a Human Intelligence Task (HIT) and can target individuals in a particular geographic region, with a strong history of completing prior HITs on MTurk. Previous research has shown that MTurk can be used to reach low-income individuals and other hard-to-reach populations, and that the political ideology of MTurk respondents is similar to that of the general population.^5–7^

### Survey design

The study was presented as “a research survey on government programs.” The survey was restricted to adults ≥18 years and U.S. residents. Eligibility criteria also included self-report of receiving SNAP benefits in the past 12 months, or an affirmative response to the USDA food insufficiency screener in the past 12 months. Of the 1179 responses, 570 met these eligibility criteria, of whom 202 were SNAP participants and 368 were nonparticipants. Duplicate responses from the same IP address were removed. The study was considered exempt by the University of Michigan Institutional Review Board—Health Sciences and Behavioral Sciences.

This survey assessed opinions on federal SNAP spending and policies to change the program's eligibility or nutritional impact. Questions were chosen to understand general support for the program, as well as policies that have been proposed previously or during the 2018 Farm Bill debate. All proposals were rated on a four-point Likert scale: strongly support, somewhat support, somewhat oppose, and strongly oppose. The exact wording of the questions and response options are provided in Table 1. The survey then included a description of America's Harvest Box proposal: “In February 2018, the Trump administration proposed replacing half of SNAP benefits with a box of shelf-stable foods, including canned fruits and vegetables, milk, pasta, cereals, and peanut butter (also known as the Harvest Box).” A close-ended question followed about whether the respondent would support the proposal and an open-ended question asked the respondent to share any additional thoughts or to elaborate on their response to support or oppose the proposal. The survey also assessed the respondent's demographics, political party affiliation, household food security status (using the USDA 6-Item Short Form Food Security Survey Module), and household SNAP participation. Questions on general and nutrition-specific policies used identical wording as previous surveys.^8–10^

**Table 1.** Support for General and Nutrition Policies to Improve the Impact of Supplemental Nutrition Assistance Program by Supplemental Nutrition Assistance Program Participation Status (*n*=570)

	Overall		SNAP participants (*n*=202)		Nonparticipants (*n*=368)		
	*n*	%	*n*	%	*n*	%	*p*^a^
General policies							
Have stricter eligibility requirements for SNAP							<0.001
Strongly support	107	18.8	23	11.4	84	22.8	
Somewhat support	146	25.6	40	19.8	106	28.8	
Somewhat oppose	201	35.3	79	39.1	122	33.2	
Strongly oppose	116	20.4	60	29.7	56	15.2	
Allow low-income college students (enrolled in higher education) to access SNAP benefits							<0.001
Strongly support	252	44.2	112	55.5	140	38	
Somewhat support	201	35.3	0	24.8	151	41	
Somewhat oppose	72	12.6	29	14.4	43	11.7	
Strongly oppose	45	7.9	11	5.5	34	9.2	
Extend the 3-month limit for able-bodied adults without dependents to receive SNAP without working							0.34
Strongly support	180	31.6	72	35.6	108	29.4	
Somewhat support	185	32.5	63	31.2	122	33.2	
Somewhat oppose	123	21.6	37	18.3	86	23.4	
Strongly oppose	82	14.4	30	14.9	52	14.1	
Nutrition policies							
Providing additional money to SNAP participants that can only be used on fruits, vegetables, or other healthful foods							0.001
Strongly support	282	49.5	122	60.4	160	43.5	
Somewhat support	186	32.6	51	25.3	135	36.7	
Somewhat oppose	64	11.2	16	7.9	48	13.0	
Strongly oppose	38	6.7	13	6.4	25	6.8	
Removing sugary drinks (such as soda) from the list of foods that can be purchased using SNAP benefits							<0.001
Strongly support	226	39.7	58	28.7	168	45.7	
Somewhat support	139	24.4	49	24.3	90	24.5	
Somewhat oppose	128	22.5	54	26.7	74	20.1	
Strongly oppose	77	13.5	41	20.3	36	9.8	
Both removing sugary drinks from SNAP and providing additional money for fruits, vegetables, or other healthful foods							0.04
Strongly support	217	38.1	71	35.2	146	39.7	
Somewhat support	158	27.7	49	24.3	109	29.6	
Somewhat oppose	124	21.8	47	23.3	77	20.9	
Strongly oppose	71	12.5	35	17.3	36	9.8	
Increase the total amount of SNAP benefits to guarantee enough to eat and good nutrition							<0.001
Strongly support	259	45.4	119	58.9	140	38.0	
Somewhat support	181	31.8	51	25.3	130	35.3	
Somewhat oppose	81	14.2	24	11.9	57	15.5	
Strongly oppose	49	8.6	8	4.0	41	11.1	
Provide more nutrition education or cooking classes to SNAP participants							0.04
Strongly support	190	33.3	59	29.2	131	35.6	
Somewhat support	240	42.1	83	41.1	157	42.7	
Somewhat oppose	89	15.6	33	16.3	56	15.2	
Strongly oppose	51	9.0	27	13.4	24	6.5	
Require all SNAP participants to receive nutrition education							<0.001
Strongly support	140	24.6	41	20.3	99	26.9	
Somewhat support	214	37.5	64	31.7	150	40.8	
Somewhat oppose	132	23.2	50	24.8	82	22.3	
Strongly oppose	84	14.7	47	23.3	37	10.1	
Harvest Box proposal							<0.001
Strongly support	79	13.9	9	4.5	70	19.0	
Somewhat support	149	26.1	33	16.3	116	31.5	
Somewhat oppose	108	19.0	43	21.3	65	17.7	
Strongly oppose	234	41.1	117	57.9	117	31.8	

^a^ *p*-Values from chi-square tests on differences in support for various policies by SNAP participation status.

SNAP, Supplemental Nutrition Assistance Program.

### Statistical analysis

Close-ended questions were analyzed using response frequencies and proportions. Variation by SNAP participation was examined using chi-square tests. Statistical analyses were performed using Stata/SE version 12. The open-ended question was analyzed for thematic content using a general inductive approach. Both authors coded all open-ended responses independently and then cross-checked their results. The themes reported were the ones that were most often discussed across all survey respondents.

## Results

Survey respondents came from 49 states across the United States. Approximately 50% of respondents were between 18 and 34 years. The majority of respondents were women (59%), white (75%), and lived with children <18 years (52%). With respect to political affiliation, 26% identified as Republican, 39% as Democrat, and 35% as Independent. Approximately 35% of respondents received SNAP benefits in the past 12 months (e.g., SNAP participants). Compared with nonparticipants, SNAP participants were more likely to live in a household with children and report higher levels of food insecurity.

Overall, 66% of respondents supported increasing federal spending for SNAP, 22% thought SNAP spending should be kept the same, and 12% believed SNAP spending should be decreased. The majority of respondents supported extending the 3-month limit for able-bodied adults without dependents to receive SNAP without working (SNAP participants 67%; nonparticipants 63%), opposed implementing stricter eligibility requirements for SNAP participation (SNAP participants 69%; nonparticipants 48%), and supported expanding SNAP benefits to low-income college students (SNAP participants 80%; nonparticipants 79%; Table 1).

With respect to strategies to improve the nutritional impact of SNAP, the majority of respondents supported financial incentives for fruits, vegetables, or other healthful foods (SNAP participants 86%; nonparticipants 80%); removing sugary drinks from products allowed under SNAP (SNAP participants 53%; nonparticipants 70%); increasing total benefits (SNAP participants 84%; nonparticipants 73%); and more nutrition education or cooking classes (SNAP participants 70%; nonparticipants 78%). The largest gap in support was in removing sugary drinks from SNAP; however, this gap narrowed when this proposal was paired with providing incentives for healthful foods (SNAP participants 60%; nonparticipants 69%).

Sixty percent of respondents opposed the America's Harvest Box proposal. Opposition differed significantly by SNAP participation status (SNAP participants 79%, nonparticipants 50%). Thematic analysis of an open-ended question regarding the Harvest Box included concerns about healthfulness of the foods provided; inability to support dietary restrictions, individual health needs or food allergies; preservation of choice; food waste; and increases in food insecurity (data not shown), all of which were raised by both SNAP participants and nonparticipants.

## Discussion

In this study, SNAP participants and nonparticipants supported increasing federal funding for SNAP and policies to expand SNAP eligibility. This study also provides some of the first reactions to policies initially proposed during the 2018 Farm Bill debate, including support to extend the time limit for SNAP participants without dependents to receive benefits without working, and to extend SNAP eligibility to low-income college students, a group facing barriers to SNAP despite high rates of food insecurity.^11^

Study findings also reiterated broad support for strategies to improve the nutritional impact of SNAP. Although strategies to increase total SNAP benefits, provide financial incentives for fruits and vegetables, and provide more nutrition education are policies that are generally supported across stakeholder groups,^12^ the proposal to remove sugary beverages from the list of foods eligible for purchase with SNAP benefits has generated heated debate between antihunger advocates and public health researchers.^13^ Currently, the USDA has denied all requests by states and municipalities to remove sugary drinks and other non-nutritious foods from SNAP arguing that these policies are difficult to implement. Although the ethics and logistics of this strategy will continue to be debated, a recent study estimated that removing sugary beverages would reduce obesity by 2.4% and type 2 diabetes incidence by 1.7% among SNAP participants, with primary benefits to minority racial/ethnic adults.^14^ Consistent with prior research,^8–10,15^ a majority of SNAP participants in this study supported removing sugary drinks from the list of SNAP-eligible foods, and this level increased when combined with financial incentives for fruits and vegetables. To date, only one study has tested the impact of these policies on nutritional outcomes in an experimental setting. In 2016, Harnack et al. conducted a randomized trial among low-income nonparticipants and found favorable improvements in energy intake and overall diet quality among adults in the “incentives plus restrictions” condition when compared with adults in the control group.^16^ More research is needed to examine the impact of these policies on food security and health outcomes as well to understand how these policies might fit into the current context of SNAP.

This was the first study to assess support for the America's Harvest Box proposal. The USDA has estimated this proposal to save $129.2 billion for the next decade.^3^ However, antihunger advocates, media outlets, and even supermarkets have expressed concern about this radical change to food distribution.^17–20^ Unlike other suggested policies, the vast majority of SNAP participants in this study disapproved of the proposal, citing concerns related to individual dietary needs, lower quality of foods, delivery logistics, and preservation of choice. Moving forward, the opinions of SNAP participants and adults at risk for food insecurity need to be represented in discussions around improving the program.

This primary limitation of this study is the generalizability of the findings, given that respondents were recruited from TurkPrime. Although past research has shown that data obtained from Internet surveys are reliable and the results of this study are similar to results from prior polls, more research is needed to confirm the results of this study using diverse methodologies.^8,10^ Another limitation is that SNAP participation was self-reported, which may have resulted in some SNAP participants being misclassified as nonparticipants. Finally, although the questions in this survey have been used in prior research, validation studies are needed to ensure that survey respondents understand what is meant by the various policy proposals and can express their support or opposition accordingly.

Despite the significant impact that altering federal food policies will have on the health and well-being of low-income Americans, there is limited research on how these changes will be received by program participants and individuals likely to participate in SNAP in the future. Proposals that change the implementation of SNAP should be rigorously tested for acceptance, feasibility, and effectiveness within this population.

## Abbreviations Used

HIT: Human Intelligence Task
SNAP: Supplemental Nutrition Assistance Program
USDA: U.S. Department of Agriculture
